# Dorsal raphe serotonin neurons inhibit operant responding for reward via inputs to the ventral tegmental area but not the nucleus accumbens: evidence from studies combining optogenetic stimulation and serotonin reuptake inhibition

**DOI:** 10.1038/s41386-018-0271-x

**Published:** 2018-11-12

**Authors:** Caleb J. Browne, Andrew R. Abela, Duong Chu, Zhaoxia Li, Xiaodong Ji, Evelyn K. Lambe, Paul J. Fletcher

**Affiliations:** 1 Department of Psychology, University of Toronto, 100 St. George Street, Toronto, ON M5S 3G3 Canada; 2 Section of Biopsychology, Campbell Family Mental Health Research Institute, Centre for Addiction and Mental Health, 250 College Street, Toronto, ON M5T 1R8 Canada; 3 Department of Psychiatry, University of Toronto, Toronto, Canada; 4 Department of Physiology, University of Toronto, Toronto, Canada; 5 Department of Obstetrics and Gynecology, University of Toronto, Toronto, Canada; 6 Present Address: Department of Neuroscience, Friedman Brain Institute, Icahn School of Medicine at Mount Sinai, New York, NY USA

**Keywords:** Motivation, Addiction, Reward, Depression, Transporters in the nervous system

## Abstract

The monoamine neurotransmitter serotonin (5-hydroxytryptamine; 5-HT) exerts an inhibitory influence over motivation, but the circuits mediating this are unknown. Here, we used an optogenetic approach to isolate the contribution of dorsal raphe nucleus (DRN) 5-HT neurons and 5-HT innervation of the mesolimbic dopamine (DA) system to motivated behavior in mice. We found that optogenetic stimulation of DRN 5-HT neurons enhanced downstream 5-HT release, but this was not sufficient to inhibit operant responding for saccharin, a measure of motivated behavior. However, combining optogenetic stimulation of DRN 5-HT neurons with a low dose of the selective serotonin reuptake inhibitor (SSRI) citalopram synergistically reduced operant responding. We then examined whether these effects could be recapitulated if optogenetic stimulation specifically targeted 5-HT terminals in the ventral tegmental area (VTA) or nucleus accumbens (NAc) of the mesolimbic DA system. Optogenetic stimulation of 5-HT input to the VTA combined with citalopram treatment produced a synergistic decrease in responding for saccharin, resembling the changes produced by targeting 5-HT neurons in the DRN. However, this effect was not observed when optogenetic stimulation targeted 5-HT terminals in the NAc. Taken together, these results suggest that DRN 5-HT neurons exert an inhibitory influence over operant responding for reward through a direct interaction with the mesolimbic DA system at the level of the VTA. These studies support an oppositional interaction between 5-HT and DA systems in controlling motivation and goal-directed behavior, and have important implications for the development and refinement of treatment strategies for psychiatric disorders such as depression and addiction.

## Introduction

Acquisition of beneficial stimuli such as food, water, or sexual partners depends on the ability of such stimuli to elicit appetitive behaviors—a process known as incentive motivation. Incentive motivation is coordinated by interactions among several neurotransmitter systems in the brain, and many lines of evidence suggest that the serotonin (5-hydroxytryptamine; 5-HT) system plays a particularly important role. For example, enhancing whole-brain 5-HT activity reduces feeding behavior [1], operant responding for food [2, 3], brain stimulation reward [4], drug self-administration [5], and appetitive behavior elicited by reward-associated stimuli [6–8]. The effects of 5-HT on motivated behavior may be mediated by neurons originating in the brainstem dorsal raphe nucleus (DRN), given that lesions or pharmacological inhibition of these neurons induces feeding [9], increases the efficacy of brain stimulation reward [10], and supports the formation of a conditioned place preference [11]. Although these inactivation studies suggest that DRN 5-HT neurons have an inhibitory role in motivated behavior, the downstream targets mediating this effect have not been identified.

Serotonin may modulate incentive motivation through interactions with the mesolimbic dopamine (DA) system [12–14]. The mesolimbic DA system is comprised of DA neurons originating in the ventral tegmental area (VTA) which predominantly terminate in the nucleus accumbens (NAc). This circuit is critical for reward processing and motivation [15–17]; enhancing mesolimbic DA activity reinforces operant behavior [18, 19], while reducing DA activity inhibits reward-related behaviors [20–22]. Given that both the VTA and NAc receive extensive serotonergic innervation [23], 5-HT neurons are ideally positioned to modulate mesolimbic DA activity at the level of DA cell bodies and their terminals, respectively. In fact, serotonin has been shown to reduce DA-dependent behavior [24–27], presumably by suppressing mesolimbic DA system activity [28–32]. Thus, 5-HT may inhibit motivated behavior by opposing mesolimbic DA system function via interactions with the VTA and NAc.

Here, we use a combined optogenetic and pharmacological approach to identify a potential pathway through which 5-HT may inhibit incentive motivation. We first examined whether optogenetic stimulation of 5-HT output from the DRN altered operant responding for the primary reinforcer saccharin as a measure of incentive motivation. In contrast to the effects of acute, pharmacological enhancement of whole-brain 5-HT via blockade of 5-HT reuptake [8], we found that optogenetic stimulation of DRN 5-HT neurons had no effect on responding for saccharin. However, when combined with the selective serotonin reuptake inhibitor (SSRI) citalopram, optogenetic stimulation of DRN 5-HT neurons synergistically reduced responding for saccharin. This decrease in responding was recapitulated by targeting 5-HT terminals in the VTA, but not in the NAc. These findings suggest that DRN 5-HT neurons inhibit operant responding for reward through an interaction with the VTA.

## Materials and methods

### Subjects

**Fig. 1:**
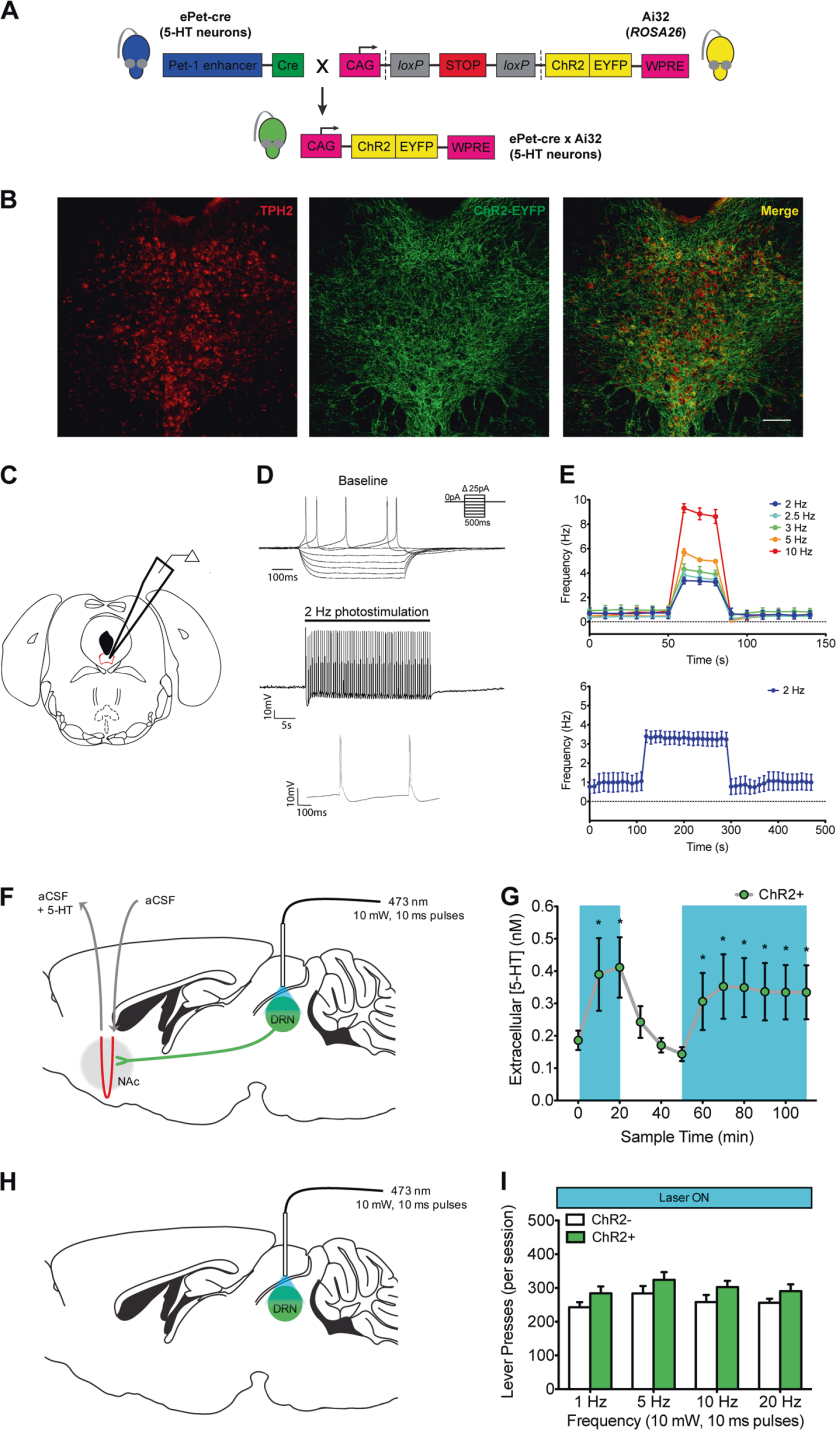
Optogenetic stimulation causes dorsal raphe nucleus (DRN) 5-HT neuron activation and enhances downstream 5-HT release, but does not alter operant responding for reward. **a** Expression of ChR2-EYFP specifically in 5-HT neurons was achieved by crossing Ai32^+/+^ mice and ePet-cre^+/−^ mice. **b** Representative coronal section showing ChR2-EYFP expression in the DRN: left, 5-HT neurons immunostained for tryptophan hydroxylase-2, a marker of 5-HT neurons; middle, cells expressing ChR2-EYFP; right, merge of tryptophan hydroxylase-2 and ChR2-EYFP channels. Scale bar represents 100 μm. **c** Schematic for ex vivo patch-clamp recordings of ChR2-EYFP expressing DRN neurons. **d** Characteristic electrophysiological response of an EYFP-positive serotonergic DRN neuron to current steps and 2 Hz photostimulation trains. **e** Top: average (±SEM) firing frequencies of DRN 5-HT neurons (*n* = 13) elicited by 30 s trains of optogenetic stimulation (2–10 Hz). Bottom: Averaged trace illustrating that extended photostimulation (2 Hz, 200 s) can maintain a significantly elevated firing frequency compared to baseline (paired-samples *t*-test: *t*_(7)_ = 5.52, *p* < 0.001), when tested in a subset of DRN 5-HT neurons. **f** Schematic for in vivo microdialysis with optogenetic stimulation of ChR2-EYFP expressing DRN 5-HT neurons. **g** Average (±SEM) extracellular 5-HT concentration within the NAc from anesthetized ChR2+ mice (*n* = 5) during periods of photostimulation (blue shaded areas) or no photostimulation (unshaded areas). *p < 0.05 vs. previous baseline sample from post hoc analysis. **h** Schematic for optogenetic stimulation (473 nm light, 10 mW, 10 ms pulses) of DRN 5-HT neurons throughout tests of operant responding for saccharin. **i** Optogenetic stimulation of DRN 5-HT neurons does not alter operant responding for saccharin: mean lever presses (±SEM) for saccharin was not different between ChR2− (*n* = 10; white bars) and ChR2+ mice (*n* = 11; green bars) at any photostimulation frequency tested (Group × Frequency: *F*_(3,57)_ = 0.06, ns; Group: *F*_(3,57)_ = 2.94, ns; independent-samples *t*-tests: all *t*_(19)_ < 1.57, all *p* > 0.066)

To express the excitatory opsin channelrhodopsin2 (ChR2) in 5-HT neurons, Ai32^+/+^ mice (Jackson 012569; [33]) were crossed with ePet-Cre^+/^^−^ mice (Jackson 012712; Fig. 1a). The ePet domain is specific to 5-HT neurons, and has been used to direct expression of ChR2 in 5-HT neurons [34–36]. Experimental mice were Ai32^+/+^ and either ePet-Cre^+/−^ (ChR2+) or ePet-Cre negative (ChR2−). ChR2+ mice exhibited expression of ChR2-EYFP in raphe 5-HT neurons (Fig. 1b), as others have observed [37]; no EYFP-positive cells were observed in ChR2− mice (data not shown). Experiments were performed on adult (10+ weeks old) male mice, unless otherwise specified. All mice were group-housed in temperature and humidity controlled environments on a 12 h light/dark cycle (lights on at 7:00 am) with food available *ad libitum*. Following surgery, mice were single-housed. In tests of responding for saccharin, mice received 2 h of limited water access each day. In all other experiments, water was available *ad libitum*. Experiments adhered to the Canadian Council of Animal Care standards and followed protocols approved by the Centre for Addiction and Mental Health Animal Care Committee and the University of Toronto Faculty of Medicine Animal Care Committee.

### Drugs

Citalopram HBr (Toronto Research Chemicals, Toronto, Canada) was dissolved in 0.9% saline, and injected intraperitoneally in a volume of 10 ml/kg. Citalopram was injected 20 min prior to behavioral testing with dose order determined by a Latin square and all test sessions separated by 72 h. Doses are expressed in terms of the free base, and were determined from previous work in our laboratory [8].

### Ex vivo electrophysiology

Coronal brain slices (400 μm) containing the DRN were obtained for all experiments using a vibratome (Dosaka Pro-7 Linear Slicer; SciMedia) in ice-cold oxygenated sucrose-substituted artificial cerebrospinal fluid (aCSF). The slices were recovered at 30 °C for a minimum of 2 h in aCSF solution containing: 128 mM NaCl, 26 mM NaHCO_3_, 10 mM d-glucose, 2 mM CaCl_2_, 2 mM KCl, 2 mM MgSO_4_, 1.25 mM NaH_2_PO_4_, pH 7.4 and saturated with 95% O_2_/5% CO_2_. l-Tryptophan (2.5 μM; Sigma-Aldrich) was included during the recovery and recording period to maintain 5-HT synthesis [38]. Patch-clamp recording was performed in l-tryptophan aCSF oxygenated with 95% O_2_/5% CO_2_ at 30° C flowing at 3–4 mL/min. Patch pipettes (2–5 MΩ) contained solution with: 120 mM potassium gluconate, 5 mM KCl, 10 mM Na_2_-Phosphocreatine, 10 mM HEPES buffer, 2 mM MgCl_2_, 120 mM potassium gluconate, 4 mM K_2_-ATP, 0.4 mM Na_2_-GTP (adjusted to pH 7.3 with KOH). DRN neurons were visualized using a fixed-stage microscope (Olympus BX50WI), and 5-HT neurons were targeted based on EYFP expression. Whole-cell recordings were performed in current-clamp with a Multiclamp 700B amplifier using pClamp10.2 software (Molecular Devices). Optogenetic stimulation (10 ms pulses; 2–10 Hz) was performed using a microscope-mounted collimated LED (473 nm, Thorlabs; 2.5 mW at the slice). Citalopram (1 μM) was bath applied in l-tryptophan-containing aCSF to determine effects on membrane properties and optogenetic stimulation response. The citalopram concentration was chosen to be within the range of citalopram that reaches the brain following a 1–10 mg/kg systemic injection [39, 40].

### In vivo microdialysis and high-performance liquid chromatography

Microdialysis procedures were carried out as previously described [41] and detailed in Supplementary Methods 1.1. Throughout the procedure, mice were maintained under inhaled isoflurane anesthesia (2%) with body temperature held at 37 °C. Mice were mounted on a stereotaxic frame, and burr holes were made in the skull above the DRN and left NAc. An optical fiber (200 µm core, 0.39 NA) connected to a 473 nm laser was positioned above the DRN (Interaural: A/P −0.8, M/L 0, D/V +3.0). Laser output was controlled by a waveform generator. A microdialysis probe (2 mm cuprophane membrane) was lowered into the NAc (Bregma: A/P + 1.5, M/L −0.7, D/V −5.0 from probe tip). The NAc was chosen as a target to sample 5-HT release following optogenetic stimulation because it receives dense input from the DRN, and is a large enough region in the mouse brain to easily accommodate a microdialysis probe. The probe was continuously perfused with aCSF at 1 µl/min. Sampling began 90 min following probe insertion. Samples were collected every 10 min, and immediately analyzed for 5-HT concentration via high-performance liquid chromatography. Baseline 5-HT concentration was considered stable when three consecutive samples varied less than 10%. Experimental procedures are detailed in Supplementary Methods 1.2.

### Behavioral testing

Responding for saccharin was measured in operant conditioning boxes (22 × 18 × 13 cm; Med Associates, St Albans, Vermont). On the front wall of the chamber, a reinforcer magazine containing an infrared photodetector centered 2.5 cm above the floor. A motor-driven dipper could be raised to deliver 0.02 ml of liquid through a hole in the magazine floor. One retractable lever was located on the left of the magazine, which was extended for the duration of operant testing. Operant boxes were illuminated by a houselight, and enclosed in a sound-attenuating chamber equipped with a ventilation fan. Mice could be tethered to optical patch cables through a hole in the roof of the operant box, which was connected in series to an optical commutator and a 473 nm laser controlled by a waveform generator (See Supplementary Methods 2.1).

Responding for saccharin was carried out as previously described [8], and detailed in Supplementary Methods 2.2. Mice were maintained on water restriction throughout testing. In 40-min sessions, mice were first trained to lever press for 0.02 ml saccharin (0.2% w/v) presented in a dipper for 5 s on a fixed ratio 1 schedule of reinforcement followed by a random ratio 4 (RR4) schedule of reinforcement (1-in-4 chance of response being rewarded). After stabilization of responding on the RR4 schedule, mice underwent stereotaxic surgery (detailed in Supplementary Methods 2.3) to implant optical fibers above the DRN (midline), VTA (bilateral), or NAc (bilateral). One week following surgery, responding for saccharin was examined in 20-min sessions with mice tethered to the optical commutator. Mice first received 10 re-training sessions, wherein no photostimulation was applied, to learn to accommodate the tether inside the operant chamber. In subsequent tests, photostimulation was applied for the duration of testing, unless otherwise specified. Experimental procedures are detailed in Supplementary Methods 2.4–2.6.

Locomotor activity was assessed as previously described [42] under dim lighting using 50 × 50 × 35 cm plexiglass chambers with an array of 11 externally mounted photobeams (Med Associates). Activity counts were recorded as horizontal photobeam breaks. Serotonin syndrome measurements were carried out under bright lighting in a 25 × 35 × 25 cm plexiglass chamber with a side-mounted camera. Procedures are described in Supplementary Methods 2.5.

### Histology

Immunohistochemistry was performed on brains collected from ChR2+ and ChR2− mice (see Supplementary Methods 3.1). Primary antibodies used were mouse monoclonal anti-TPH2 (1:500; Sigma-Aldrich) and chicken anti-GFP (1:1000; Abcam). Secondary antibodies used were donkey anti-mouse Alexa 594 (1:1000; Invitrogen) and donkey anti-chicken Alexa 488 (1:1000; Jackson Immunoresearch). Sections were imaged using a confocal microscope.

### Statistics

Data were analyzed using Statistica v13 (TIBCO, Palo Alto, CA). Electrophysiology and serotonin syndrome data were analyzed using paired and independent-samples *t*-tests. Neurochemical data were analyzed using one-way ANOVAs with Sample as a within-subjects factor. Behavioral testing data with photostimulation alone were analyzed using independent-samples *t*-tests, or one-way ANOVAs comparing each photostimulation session with previous and subsequent “OFF” sessions. Behavioral testing data that combined photostimulation and citalopram treatment were analyzed using mixed-model two or three-way ANOVAs, depending on experimental design, with Group (ChR2− and ChR2+) as a between-subjects factor, and Laser (ON or OFF), Citalopram (vehicle, 5, or 10 mg/kg), and Time (across a session) as within-subjects factors. Post hoc analyses were performed using Fisher’s LSD test.

## Results

### Optogenetic stimulation increases DRN 5-HT neuron activity and causes downstream 5-HT release

Ex vivo patch-clamp electrophysiology experiments (Fig. 1c) confirmed that ChR2-EYFP-expressing 5-HT neurons (*n* = 13 from four ChR2+ mice) were reliably depolarized by 10 ms pulses of 473 nm blue light, which frequently elicited doublets (Fig. 1d; Table S1). These neurons easily followed 30-s stimulation trains at different frequencies (2–10 Hz; Fig. 1e, top panel). Moreover, labeled 5-HT neurons reliably sustained action potential firing in response to prolonged photostimulation trains delivered at 2 Hz (Fig. 1e, bottom panel; paired-samples *t*-test: *t*_(7)_ = 5.52, *p* < 0.001) and 3 Hz (data not shown). In vivo microdialysis experiments in anesthetized female ChR2+ mice (*n* = 6) confirmed that optogenetic stimulation of DRN 5-HT neurons increased downstream 5-HT release (Fig. 1f). Photostimulation (2.5 Hz, 10 mW, 10 ms pulses) for 20 min enhanced extracellular 5-HT levels (Fig. 1g; Sample: *F*_(7, 40)_ = 7.49, *p* < 0.001; Post hoc: *p* < 0.001 for samples 2 and 3 vs. sample 1). Thirty minutes later, photostimulation was reapplied for 60 min. During this time, 5-HT levels increased to a plateau within 20 min (Fig. 1g; Samples 6–12; Sample: *F*_(6,28)_ = 5.24, *p* < 0.01; Post hoc: *p* < 0.01 for samples 7–12 vs. sample 6), consistent with previous reports that 5-HT release can be sustained for extended periods of time [43].

### Optogenetic stimulation of DRN 5-HT neurons does not alter responding for a primary reinforcer

We next investigated the effects of optogenetic stimulation (10 mW, 10 ms pulses) of DRN 5-HT neurons on responding for saccharin in ChR2− (*n* = 10) and ChR2+ (*n* = 11) mice (Fig. 1h). We tested different photostimulation frequencies (1, 5, 10, and 20 Hz) chosen to represent different firing frequencies of DRN 5-HT neurons observed during reward-related behavior [44, 45]. However, optogenetic stimulation had no effect on responding at any frequency tested (Fig. 1i; Group × Frequency: *F*_(3,57)_ = 0.06, ns; Group: *F*_(3,57)_ = 2.94, ns; independent-samples *t*-tests: all *t*_(19)_ < 1.57, all *p* > 0.066). It is possible that responding for saccharin was unaffected by optogenetic stimulation because rapid reuptake prevents the accumulation of 5-HT typically required to induce a behavioral change. Indeed, most studies examining the involvement of 5-HT in reward-related behavior employ manipulations that impair 5-HT reuptake rather than stimulate 5-HT release, and these two manipulations produce distinct patterns of brain activity [46]. Thus, we next tested whether combining optogenetic stimulation with a low dose of the SSRI citalopram could alter responding for saccharin, which would presumably enable accumulation of 5-HT within downstream targets of the DRN.

### Combining optogenetic stimulation of DRN 5-HT neurons with citalopram treatment synergistically enhances extracellular 5-HT levels

**Fig. 2:**
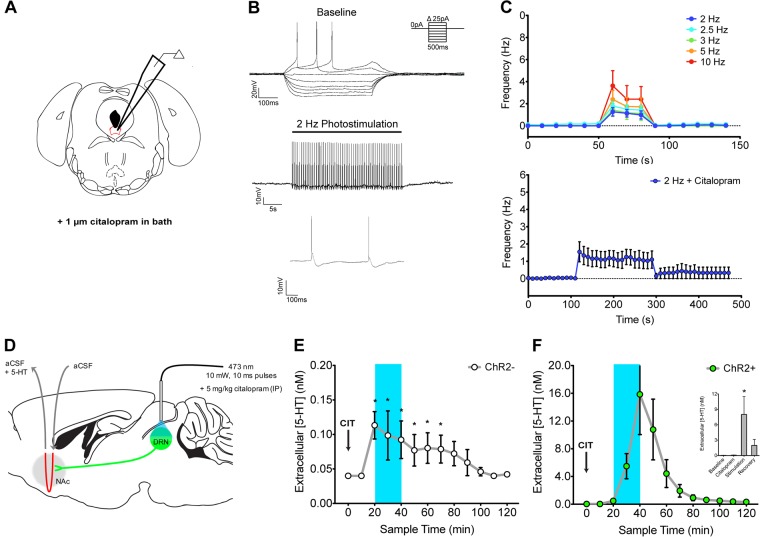
Combining optogenetic stimulation of dorsal raphe nucleus (DRN) 5-HT neurons with the SSRI citalopram synergistically enhances downstream levels of extracellular 5-HT. **a** Schematic for ex vivo patch-clamp recordings combining optogenetic stimulation ChR2-EYFP expressing DRN neurons with bath application of 1 μm citalopram. **b** Electrophysiological response of an EYFP-positive 5-HT neuron to current steps in the presence of 1 µM citalopram, and combined with 2 Hz photostimulation. **c** Top: Average (±SEM) firing frequencies of DRN 5-HT neurons (*n* = 8) elicited by 30 s trains of optogenetic stimulation with 1 μm citalopram in bath (2–10 Hz). Bottom: Averaged trace illustrates that extended photostimulation (2 Hz, 200 s) can maintain a significantly elevated firing frequency compared to baseline (paired-samples *t*-test: *t*_(5)_ = 4.4, *p* < 0.01), when tested in a subset of DRN 5-HT neurons. **d** Schematic for in vivo microdialysis experiments combining 2.5 Hz optogenetic stimulation of DRN 5-HT neurons with systemic 5 mg/kg citalopram treatment. **e** Extracellular 5-HT concentration within the NAc of anesthetized ChR2− mice (**e**; *n* = 4) and ChR2+ mice (**f**, n = 4) following intraperitoneal citalopram injection (“CIT”) and optogenetic stimulation of DRN 5-HT neurons (blue shaded area). Inset in **f** shows average 5-HT concentration within each phase of sampling for ChR2+ mice. *p < 0.05 vs. previous baseline sample

**Fig. 3:**
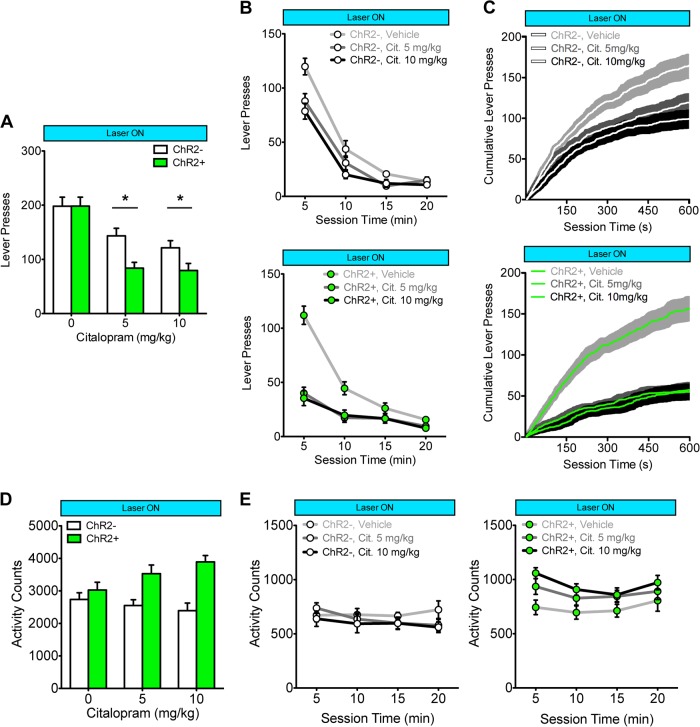
Combining optogenetic stimulation (10 mW, 10 ms pulses) of dorsal raphe nucleus (DRN) 5-HT neurons with the SSRI citalopram synergistically reduces operant responding for saccharin. **a** The combination of 2.5 Hz DRN photostimulation and citalopram treatment reduced responding to a greater extent in ChR2+ mice (*n* = 11, green bars) compared to ChR2− mice (*n* = 10, white bars; Group × Citalopram: *F*_(2,38)_ = 4.66, *p* < 0.05; *p < 0.05 between ChR2− and ChR2+). **b** Within-session data from **a**; Compared to ChR2− mice (top, white symbols), ChR2+ mice (bottom, green symbols) showed a greater decline in responding early in test sessions (Group × Citalopram × Time: *F*_(6,114)_ = 4.01, *p* < 0.001). **c** Average (±SEM, shaded area) cumulative response rate plots for the first 10 min of testing in ChR2− (top, white line) and ChR2+ mice (bottom, green line). **d** Combining DRN 5-HT neuron photostimulation with citalopram treatment increased locomotor activity (Group × Citalopram: *F*_(2,38)_ = 19.18, *p* < 0.001). **e** Within-session data from **d**; the increase in activity produced by combined photostimulation and citalopram treatment was consistent across time (Group × Citalopram × Time: *F*_(6,114)_ = 0.77, ns, Group × Citalopram: *F*_(2,38)_ = 19.20, *p* < 0.001). Data are expressed as mean (±SEM)

In DRN slices from ChR2+ mice, bath application of 1 µM citalopram resulted in a decrease in firing rate in EYFP-positive neurons (*n* = 8 from three ChR2+ mice; Fig. 2a) due to the loss of doublet firing that was observed under baseline conditions. This change likely resulted from citalopram’s effects on membrane potential (baseline: −68 ± 3 mV, *n* = 13; citalopram: -81 ± 4 mV, *n* = 8; unpaired *t-*test: *t*_(19)_ = 2.6; *p* < 0.05) and input resistance (baseline: 521 ± 44 MΩ; citalopram: 340 ± 68 MΩ; *t*_(19)_ = 2.3; *p* < 0.05) (Fig. 2b; Table S1). Despite these electrophysiological changes, optogenetic stimulation elicited firing of 5-HT neurons at multiple frequencies (Fig. 2c, top panel). Further, with citalopram on board, DRN 5-HT neurons could still be entrained to fire for prolonged periods in response to 2 Hz photostimulation (Fig. 3c, bottom panel; paired-samples *t*-test: *t*_(5)_ = 4.2, *p* < 0.01) and 3 Hz photostimulation (data not shown). Changes in downstream 5-HT levels following combined DRN photostimulation (2.5 Hz, 10 mW, 10 ms pulses) and citalopram treatment were then measured in ChR2− (*n* = 4) and ChR2 + (*n* = 4) mice (Fig. 2d). In ChR2− mice, citalopram increased extracellular 5-HT concentration to a peak within 20 min which steadily declined over testing (Fig. 2e; Sample: *F*_(12,36)_ = 3.758, *p* < 0.01; Post hoc: Samples 3–8 vs. Sample 1, all *p* < 0.05); DRN photostimulation had no further effect (Post hoc: Samples 4–5 vs. Sample 3, ns). In ChR2+ mice, combined DRN photostimulation and citalopram treatment produced a large increase in 5-HT concentration during the 20 min photostimulation period, although the magnitude of this effect was highly variable (Fig. 2f; Sample 3–5: *F*_(12, 36)_ = 4.13, *p* < 0.05). Averaging data for the ChR2+ group across periods of testing (Baseline, Citalopram Treatment, Stimulation, Recovery) shows that photostimulation produced a significant increase in extracellular 5-HT (Fig. 2f inset; sampling period: *F*_(3,12)_ = 5.15, *p* < 0.05; Post hoc: Stimulation vs. Baseline, *p* < 0.05).

### Optogenetic stimulation of DRN 5-HT neurons combined with citalopram treatment synergistically reduces responding for a primary reinforcer

Combining DRN photostimulation (2.5 Hz, 10 mW, 10 ms pulses) and citalopram treatment (5, 10 mg/kg, or vehicle) synergistically reduced responding for saccharin in ChR2+ mice relative to ChR2− mice (Fig. 3a; mice from Fig. 1i; Group × Citalopram: *F*_(2,38)_ = 4.66, *p* < 0.05; post hoc: 5 and 10 mg/kg both *p* < 0.05, vehicle, ns). A within-session analysis found that this difference was particularly pronounced at the beginning of testing (Fig. 3b; Group × Citalopram × Time: *F*_(6,114)_ = 4.01, *p* < 0.001). Average cumulative response plots (Fig. 3c) demonstrate that combined DRN photostimulation and citalopram treatment greatly reduced response rates from the beginning of the session in ChR2+ (bottom panel) mice compared to ChR2− mice (top panel). This experiment was repeated using higher photostimulation frequencies, and a similar effect was observed with 5 Hz (Figure S1-A; Group × Citalopram × Time: *F*_(6,114)_ = 11.45, *p* < 0.0001) and 10 Hz (Figure S1-B; Group × Citalopram × Time: *F*_(6,114)_ = 13.79, *p* < 0.0001). In tests of locomotor activity, the combination of photostimulation and citalopram increased activity (Fig. 3d; Group × Citalopram: *F*_(2,38)_ = 19.18, *p* < 0.0001), which was consistent across time (Fig. 3e; Group × Citalopram × Time: *F*_(6,114)_ = 0.77, ns; Group × Citalopram: *F*_(2,38)_ = 19.20, *p* < 0.0001). DRN photostimulation combined with citalopram did not produce any observable signs of serotonin syndrome in ChR2+ mice (See Table S2; all *t*_(19)_ < 1.51, all *p* > 0.148).

### Optogenetic stimulation of 5-HT input to the VTA combined with citalopram treatment synergistically reduces responding for a primary reinforcer

**Fig. 4:**
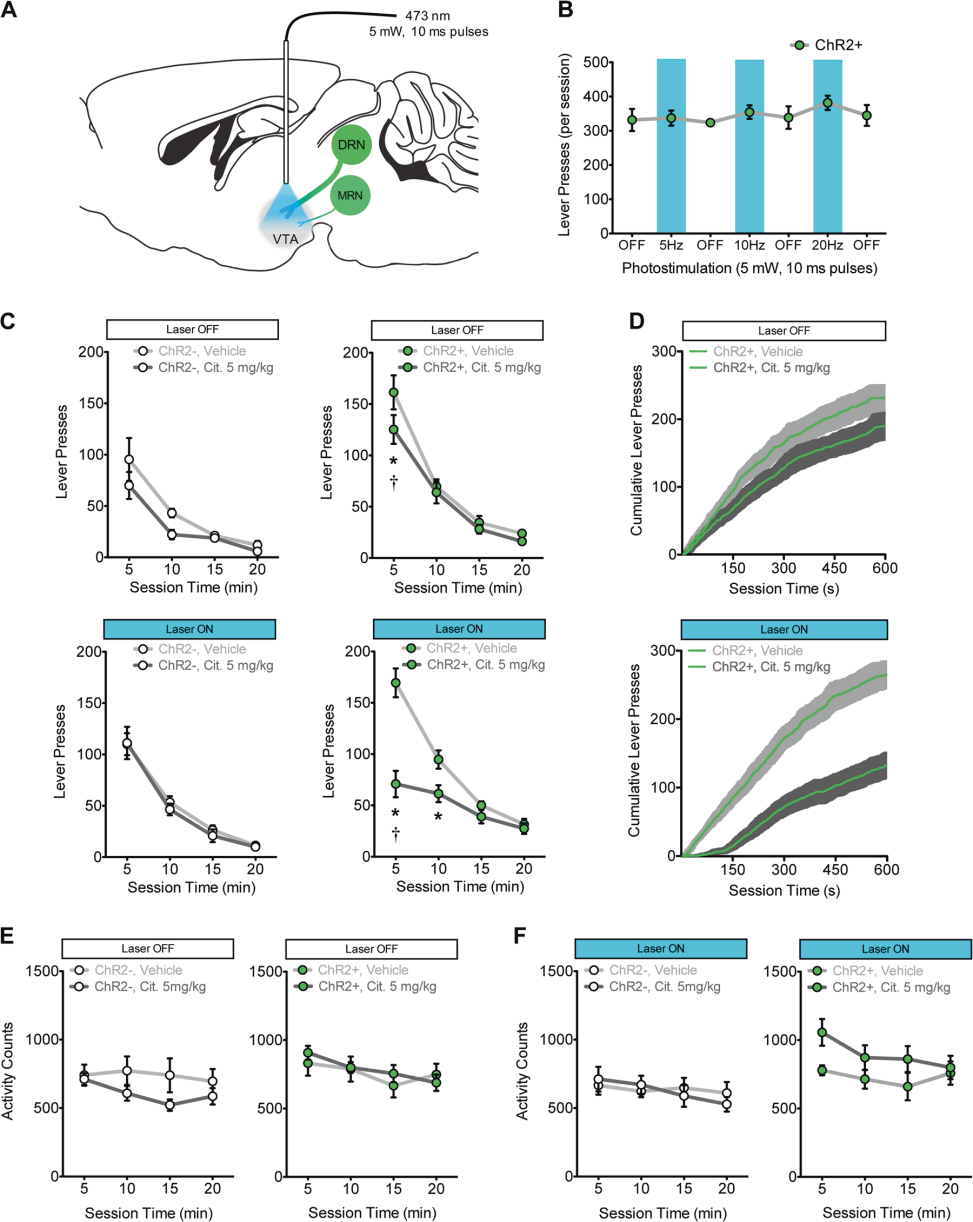
Optogenetic stimulation of 5-HT terminals in the ventral tegmental area (VTA) combined with 5 mg/kg citalopram treatment synergistically reduces operant responding for saccharin. **a** Schematic for optogenetic stimulation of 5-HT terminals in the VTA (5 mW, 10 ms pulses, 5 Hz) applied throughout tests of responding for saccharin in ChR2− mice (*n* = 5, white symbols) and ChR2+ mice (*n* = 7, green symbols). DRN, dorsal raphe nucleus; MRN, median raphe nucleus (MRN presented for completeness, weight of lines illustrates differential contribution of 5-HT inputs from DRN and MRN). **b** Photostimulation alone did not alter responding for saccharin in ChR2+ mice at any frequency tested; responding was not different between photostimulation sessions (blue shaded area) and sessions without photostimulation (“OFF”; ANOVAs across all OFF–ON–OFF periods: *F*_(1,13)_ < 1.04, *p* > 0.325). **c** Combining citalopram treatment with optogenetic stimulation of 5-HT inputs to the VTA does not significantly alter responding in ChR2− mice (left panels; Laser × Citalopram × Time: *F*_(3,12)_ = 1.43, ns), but reduced responding in a time-dependent manner in ChR2+ mice (right panels; Laser × Citalopram × Time: *F*_(3,18)_ = 5.09, *p* < 0.05; two-way ANOVA in first 5 min: Laser × Citalopram: *F*_(1,6)_ = 32.13, *p* < 0.001; †*p* < 0.001 between laser ON and OFF for 5 mg/kg citalopram; *p < 0.05 between vehicle and citalopram). **d** Average (±SEM, shaded area) cumulative response rate plots for the first 10 min of test sessions from ChR2+ mice treated with citalopram alone (top) or citalopram combined with photostimulation (bottom). **e** Citalopram alone did not differentially alter locomotor activity between ChR2− (left) and ChR2+ (right) mice (Group × Citalopram: *F*_(1,10)_ = 3.219, ns; Citalopram: *F*_(1,10)_ = 0.727, ns). **f** Combination of photostimulation with citalopram treatment produced an increase in activity that was consistent across time (Group × Citalopram × Time: *F*_(3,30)_ = 1.09, ns; Group × Citalopram: *F*_(1,10)_ = 6.80, *p* < 0.05). Data are expressed as mean (±SEM)

Following acquisition of responding for saccharin on a RR4 schedule of reinforcement, ChR2− (*n* = 5) and ChR2+ (*n* = 7) mice received bilateral optical fiber implants targeting the VTA (Fig. 4a; Figure S2A). The effect of photostimulation alone (5 mW, 10 ms pulses) was first examined in ChR2+ mice. On alternating test days, mice received either photostimulation at different frequencies (5, 10, or 20 Hz), or no photostimulation (“OFF”). Photostimulation of 5-HT terminals in the VTA alone had no effect on responding for saccharin in ChR2+ mice (Fig. 4b; ANOVAs across all OFF–ON–OFF periods: all *F*_(2,12)_ < 1.04, all *p* > 0.325). Next, both ChR2− and ChR2+ mice were tested on four occasions in a randomized order following injection of vehicle or 5 mg/kg citalopram with and without the application of 5 Hz VTA photostimulation. In ChR2+ mice (Fig. 4c, right panels), combining photostimulation of 5-HT terminals in the VTA with citalopram treatment reduced responding in a time-dependent manner (Laser × Citalopram × Time: *F*_(3,18)_ = 5.09, *p* < 0.05). Tests of simple interactions at each time bin found that within the first 5 min of testing, photostimulation combined with citalopram reduced responding to a greater extent than citalopram alone (Laser × Citalopram interaction: *F*_(1,6)_ = 32.13, *p* < 0.001; Post hoc: vehicle vs. citalopram at both photostimulation conditions *p* < 0.01, citalopram 5 mg/kg laser ON vs. OFF *p* < 0.001). Although no significant interactions were observed for the remaining time bins (Laser × Citalopram: all *F* < 2.80, all *p* > 0.14), post hoc analyses found a significant reduction in the second time bin only when photostimulation was combined with citalopram (*p* < 0.05). In ChR2− mice (Fig. 4c, left panels), no differential effects were observed between citalopram alone and citalopram combined with photostimulation (Laser × Citalopram × Time: *F*_(3,12)_ = 1.43, ns). Average cumulative response plots for ChR2+ mice (Fig. 4d) demonstrate that the effect of citalopram combined with photostimulation was driven by a reduction in response rate early on in test sessions. In tests of locomotor activity, citalopram alone did not differentially affect ChR2− and ChR2+ mice (Fig. 4e; Group × Citalopram: *F*_(1,10)_ = 3.219, ns; Citalopram: *F*_(1,10)_ = 0.727, ns). When photostimulation was combined with citalopram treatment, activity was increased consistently across time (Fig. 4f; Group × Citalopram × Time: *F*_(3,30)_ = 1.09, ns; Group × Citalopram: *F*_(1,10)_ = 6.80, *p* < 0.05).

### Optogenetic stimulation of 5-HT input to the NAc combined with citalopram treatment does not reduce responding for a primary reinforcer

**Fig. 5:**
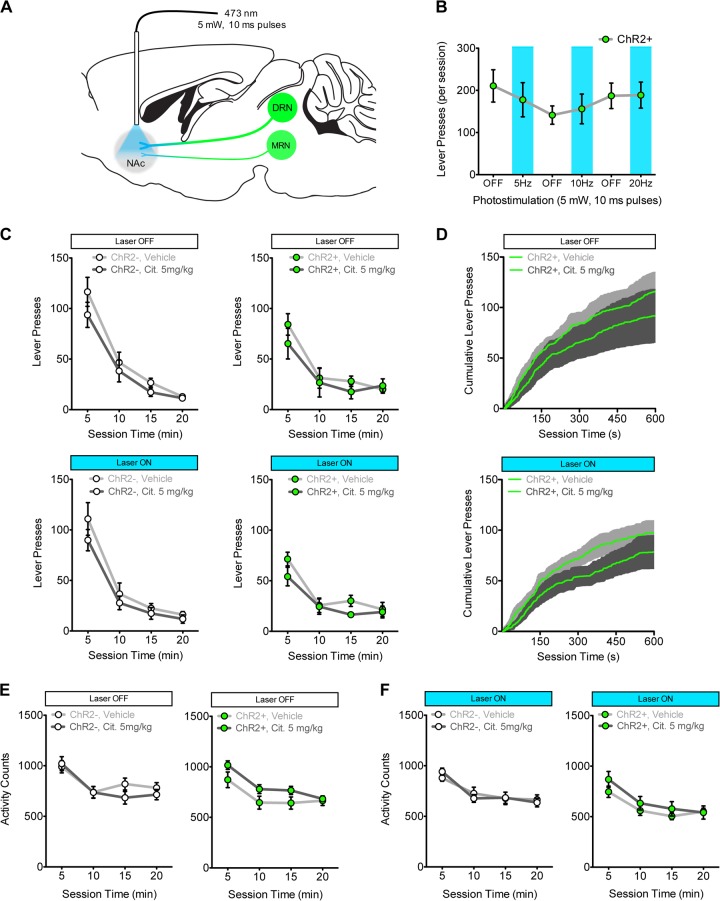
Combining optogenetic stimulation of 5-HT terminals in the nucleus accumbens (NAc) with 5 mg/kg citalopram treatment does not alter operant responding for saccharin. **a** Schematic for optogenetic stimulation of 5-HT terminals in the NAc (5 mW, 10 ms pulses, 5 Hz) applied throughout tests of responding for saccharin in ChR2− mice (*n* = 8, white symbols) and ChR2+ mice (*n* = 8, green symbols). DRN, dorsal raphe nucleus; MRN, median raphe nucleus (MRN presented for completeness, weight of lines illustrates differential contribution of 5-HT inputs from DRN and MRN). **b** Photostimulation alone did not alter responding for saccharin in ChR2 + mice at any frequency tested. Responding was not different between photostimulation sessions (blue shaded area) and sessions without photostimulation (“OFF”; ANOVAs across all OFF–ON–OFF periods: *F*_(1,15)_ < 0.25, *p* > 0.325). **c** Combining photostimulation of 5-HT terminals in the NAc with citalopram treatment did not differentially affect responding for saccharin across time in ChR2− mice (left panels; Laser × Citalopram × Time: *F*_(3,21)_ = 0.26, ns) and ChR2+ mice (right panels; Laser × Citalopram × Time: *F*_(3,21)_ = 0.68, ns). **d** Average (±SEM, shaded area) cumulative response rate plots for the first 10 min of test sessions from ChR2+ mice treated with citalopram alone (top) or citalopram combined with photostimulation (bottom). Locomotor activity was unaffected by citalopram alone (**e**; Group × Citalopram: *F*_(1,14)_ = 0.34, ns; Citalopram: *F*_(1,14)_ = 0.857, ns) or the combination of citalopram and photostimulation (**f**; Group × Citalopram × Time: *F*_(3,42)_ = 0.93, ns; Group × Citalopram: *F*_(1,14)_ = 0.02, ns). Data are expressed as mean (±SEM)

Following acquisition of responding for saccharin, ChR2− (*n* = 8) and ChR2+ (*n* = 8) mice received bilateral optical fiber implants targeting the NAc (Fig. 5a; Figure S2B). These mice were trained and tested in an identical manner to mice implanted with fibers targeting the VTA (see previous section). Photostimulation of 5-HT terminals in the NAc alone had no effect on responding in ChR2+ mice (Fig. 5b; ANOVAs across all OFF–ON–OFF periods: *F*_(2,14)_ < 0.25, ns). Combining photostimulation of 5-HT terminals in the NAc with citalopram treatment did not differentially affect responding across time for saccharin in ChR2+ mice (Fig. 5c, right panels; Laser × Citalopram × Time: *F*_(3,21)_ = 0.68, ns) or ChR2− mice (Fig. 5c, left panels; Laser × Citalopram × Time: *F*_(3,21)_ = 0.26, ns). Average cumulative response plots in ChR2+ mice (Fig. 5d) show that the combined photostimulation and citalopram condition produced a similar pattern of responding to the citalopram alone condition. Similarly, no differential effects on locomotor activity were observed between ChR2− or ChR2+ mice following citalopram alone (Fig. 5e; Group × Citalopram: *F*_(1,14)_ = 0.34, ns; Citalopram: *F*_(1,14)_ = 0.857, ns) or in combination with photostimulation (Fig. 5f; Group × Citalopram × Time: *F*_(3,42)_ = 0.93, *p* = 0.4347; Group × Citalopram: *F*_(1,14)_ = 0.02, ns).

## Discussion

The present work examines the role of 5-HT neurons originating in the DRN and their downstream inputs to the mesolimbic DA system in modulating incentive motivation. Using a combined optogenetic and pharmacological approach we demonstrate that enhancing DRN 5-HT output suppresses operant responding for the primary reinforcer saccharin, and that these effects are reproduced by selective enhancement of 5-HT input to the VTA, but not the NAc. These findings are consistent with an inhibitory role for DRN 5-HT neurons in modulating incentive motivation, and suggest that these effects may be mediated by a direct interaction with the mesolimbic DA system via inputs to the VTA.

The majority of ascending 5-HT neurons originate in the DRN [23, 47], and evidence suggests these neurons are important for modulating incentive motivation. Dorsal raphe 5-HT neurons appear to code some aspects of rewarding stimuli [44, 45, 48–50], and reducing DRN 5-HT output facilitates motivated behaviors [9–11]. However, previous studies have been unable to identify the effects of enhancing DRN 5-HT output on incentive motivation due to an inability to selectively target 5-HT neurons for activation. To overcome this, we used an optogenetic approach to stimulate genetically identified 5-HT neurons. Consistent with studies inhibiting DRN 5-HT output, optogenetic stimulation of DRN 5-HT neurons combined with a low dose of the SSRI citalopram reduced operant responding for a primary reinforcer. This effect also parallels findings that elevating whole-brain 5-HT reduces operant responding for reward, including work from our lab using similar operant testing procedures [8].

The ability of DRN 5-HT neurons to inhibit operant responding for reward may be mediated by a downstream interaction with the mesolimbic DA system. The DRN innervates both the VTA and the NAc, and 5-HT has been shown to inhibit mesolimbic DA activity. For example, global pharmacological inhibition or lesions of 5-HT neurons increases the firing rate of VTA DA neurons [28, 51], which may be mediated by 5-HT neurons originating in the DRN [29, 31]. Given the importance of the mesolimbic DA system in promoting reward-related behavior, inhibition of this system would be expected to reduce operant responding for rewards. Indeed, we show that enhancing 5-HT input to the VTA reduces operant responding for saccharin in a manner similar to enhancing DRN 5-HT output. These studies provide initial evidence that 5-HT neurons inhibit incentive motivation through a direct interaction with the mesolimbic DA system, consistent with the idea that 5-HT and DA act in opposition to coordinate goal-directed behavior [12, 13, 52, 53].

It is possible that photostimulation of 5-HT terminals in the VTA may also activate serotonergic fibers of passage within the medial forebrain bundle located ventral to the optical fiber. This may be unlikely considering that stimulation of 5-HT fibers in the NAc itself, a major terminal region of those ascending 5-HT neurons, did not alter responding for saccharin. However, this limitation applies to any optogenetic study targeting 5-HT fibers in the midbrain, and should be addressed in future studies.

Enhancing 5-HT input to the VTA reduced responding for saccharin, while enhancing 5-HT input to the NAc had no effect. Although this finding suggests that the VTA is important for mediating the effects of 5-HT on operant responding for reward, it does not preclude a modulatory role for 5-HT within the NAc. Previous studies have demonstrated that activation or blockade of 5-HT receptors in the NAc has various effects on NAc activity and reward-related behavior [54–56]. Several factors may explain the lack of effects observed here, including the specific reinforcer used, the behavior examined, or the complexity of 5-HT signaling within the NAc [57, 58]. Future studies should investigate how 5-HT signaling within the NAc, as well as the VTA, alters other behavioral measures of incentive motivation, and identify the contribution of different 5-HT receptors to these effects.

In order to confirm that photostimulation of the DRN activated 5-HT neurons, we measured extracellular levels of 5-HT in the NAc. The NAc was chosen for this purpose because it receives strong input from the DRN [47], and is a large enough structure to accommodate placement of a microdialysis probe in the mouse brain. Photostimulation of the DRN produced a small and sustained elevation of 5-HT levels, but this manipulation was not sufficient to alter responding for saccharin. It is possible that operant responding for a primary reinforcer such as saccharin may be an over-trained response, or may be a behavior that is controlled by multiple neurochemical systems which would be difficult to modify by small, naturally occurring changes in 5-HT release. Based on our previous work with citalopram, and serotonin transporter knockout mice, we suggested that 5-HT influences on incentive motivation may be more apparent with reinforcers that maintain lower levels of responding [8]. Accordingly, it would be of interest to examine whether photostimulation of 5-HT neurons, in the absence of citalopram, is sufficient to reduce responding for stimuli such as conditioned, or sensory, reinforcers.

Responding for saccharin was reduced by combining a low dose of citalopram with photostimulation of the DRN (and subsequently the VTA). This combination also increased levels of extracellular 5-HT in the NAc to a much higher degree than photostimulation alone. However, since stimulation of NAc terminals did not alter responding for saccharin, it seems unlikely that the behavioral effects of citalopram combined with DRN stimulation are mediated by this extraordinarily high level of 5-HT release. Although we did not measure 5-HT levels in the VTA due to technical limitations of sampling from this region, it is probable that 5-HT levels would also be elevated to a great extent here. The magnitude of increase in 5-HT release observed raises a concern about the physiological relevance of these neurochemical effects in relation to behavioral changes. However, reductions in responding were apparent within 5 min of testing, much earlier than the peak 5-HT release observed to be produced by this combined manipulation. Further, the differential impact of photostimulation (combined with citalopram) of the VTA vs. the NAc points to a functional difference between 5-HT inputs to these two brain areas.

The combination of citalopram treatment with optogenetic stimulation of DRN 5-HT neurons or inputs to the VTA reduced operant responding early on in test sessions. However, responding still declined further within the session, reaching similar rates of responding as observed under control conditions. This suggests that the reduction in responding is not attributable to some generalized non-specific impairment, which might be expected to induce a stable, low level of responding across the session. Consistent with this view, the combination of citalopram and optogenetic stimulation did not induce signs of the serotonin syndrome or reduce locomotor activity. In fact, a small increase in locomotor activity was observed. This effect may relate to the opposing effects of 5-HT_2A_ and 5-HT_2C_ receptors on locomotor activity in mice [59]. Given that citalopram has some 5-HT_2C_ receptor antagonist properties [60], this mild increased locomotion may result from a shift towards 5-HT_2A_-mediated signaling, facilitating locomotor activity [59]. Thus, it is unlikely that the reductions in responding for reward under these conditions resulted from general disruptions to behavioral sequencing or compromised motor function. Examination of the effects of serotonergic manipulations on feeding behavior suggest that 5-HT alters feeding motivation by enhancing the onset of satiety [61]. If a similar process were operating here, we would expect changes in responding to occur late rather than early in the session. That pattern was not observed, suggesting that a satiety-like effect does not mediate the reductions in responding. One possible explanation for the pattern of behavior observed in these studies is a reduction in incentive motivation for the primary reinforcer. We have previously hypothesized that 5-HT inhibits operant responding for multiple types of reinforcers, including saccharin, by reducing incentive motivation [8]. Consistent with this idea, informal observation of the behavior of animals in the present study showed that under conditions of combined citalopram and photostimulation, mice often made a series of responses and collected the reward before disengaging for a brief period and subsequently resuming responding. However, further studies using different types of rewarding stimuli are needed to test the hypothesis that the effects observed here are indeed a result of a 5-HT-mediated reduction in incentive motivation.

An important observation from these experiments is that behavior was altered only when optogenetic stimulation was combined with citalopram treatment, and the effects were not frequency-dependent. These findings may highlight the importance of 5-HT transporter function in regulating 5-HT neurotransmission and its behavioral consequences [43]. The 5-HT transporter tightly regulates 5-HT levels in the extracellular space and rapidly terminates 5-HT signaling [43, 62]. We show that optogenetic stimulation of DRN 5-HT neurons increases extracellular 5-HT, but this reaches a plateau (Fig. 1g), likely due to rapid reuptake by the 5-HT transporter. When the 5-HT transporter is inhibited by citalopram, extracellular 5-HT is increased (Fig. 2e) concurrent with a compensatory decrease in the DRN 5-HT neuron firing rate (Figs. 2b, c), as shown by others as well [63]. Thus, combining optogenetic stimulation with citalopram likely enabled 5-HT to accumulate, which may be required to produce the behavioral effects observed.

The present findings may help explain why serotonin-based pharmacotherapies are ineffective in treating psychiatric disorders in certain patients. Drugs that block the serotonin transporter, including the SSRIs citalopram (Celexa) and fluoxetine (Prozac), are front-line treatments for depression, obsessive-compulsive disorder, and generalized anxiety disorder. However, these compounds typically have a delayed therapeutic onset—a period that can be marked by worsening psychiatric symptoms and elevated risk of suicide [64]. Compared to non-serotonergic antidepressants, SSRIs produce side effects of avolition and sexual dysfunction—possibly reflecting motivational impairments—at higher rates [65–68]. Further, antidepressant medications that are less selective for the 5-HT system seem to be more effective in treating motivational symptoms of depression [69, 70]. It is plausible that SSRI-mediated enhancement of 5-HT neurotransmission dampens behavioral responses to rewarding stimuli through an interaction with mesolimbic DA system activity, which may lead to exacerbation of pathological symptoms in the short term.

In conclusion, the present studies suggest that DRN 5-HT neurons exert an inhibitory influence over incentive motivation for a primary reward, and point towards a DRN–VTA pathway mediating this effect. Future studies should more extensively characterize this 5-HT–DA interaction in the context of other behavioral measures of incentive motivation and identify the receptor mechanisms involved. A better understanding of how 5-HT and DA interact to control incentive motivation will help uncover fundamental mechanisms of reward processing and motivation, which are critical for refining treatment strategies for psychiatric illness.

## Funding and disclosure

This work was supported by an operating grant (MOP-13628) from the Canadian Institutes of Health Research (CIHR) to P.J.F., a Canada Research Chair in Developmental Cortical Physiology to E.K.L., a Discovery Grant from the Natural Science and Engineering Research Council of Canada (NSERC) to E.K.L., a CIHR postdoctoral fellowship A.R.A., and a Doctoral Scholarship to C.J.B. The authors declare no competing interests.

## Electronic supplementary material

Figure S1

Figure S2

Supplementary Material

Supplementary Material ReadMe File
