# Supplementary figures and images for: Dorsal raphe serotonin neurons inhibit operant responding for reward via inputs to the ventral tegmental area but not the nucleus accumbens: evidence from studies combining optogenetic stimulation and serotonin reuptake inhibition
Source: Neuropsychopharmacology. 2018 Nov 12;44(4):793–804. doi: 10.1038/s41386-018-0271-x (PMC6372654; doi:10.1038/s41386-018-0271-x)

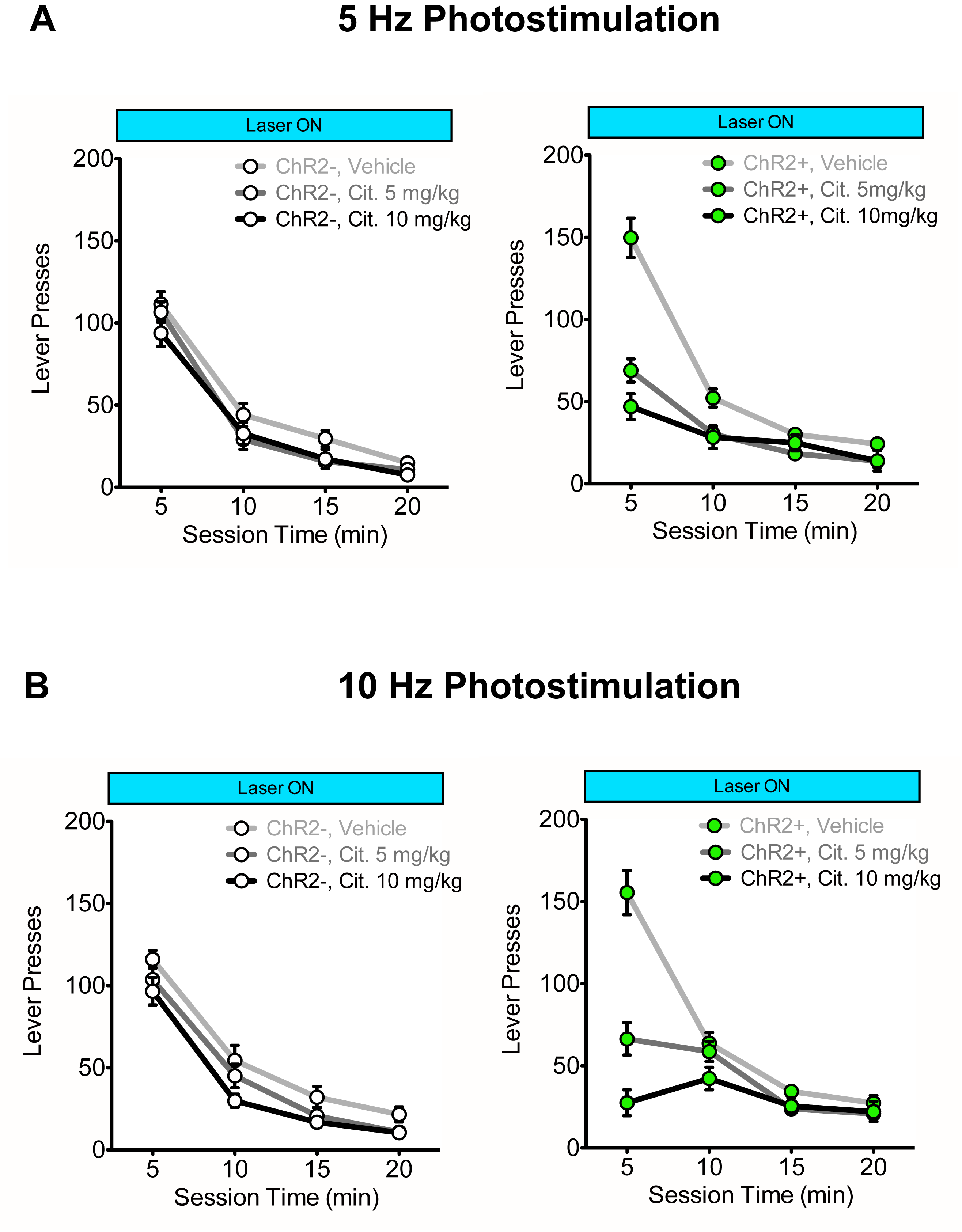

Supplement: Supplementary file 1 — Figure S1 [file 41386_2018_271_MOESM1_ESM.tif]

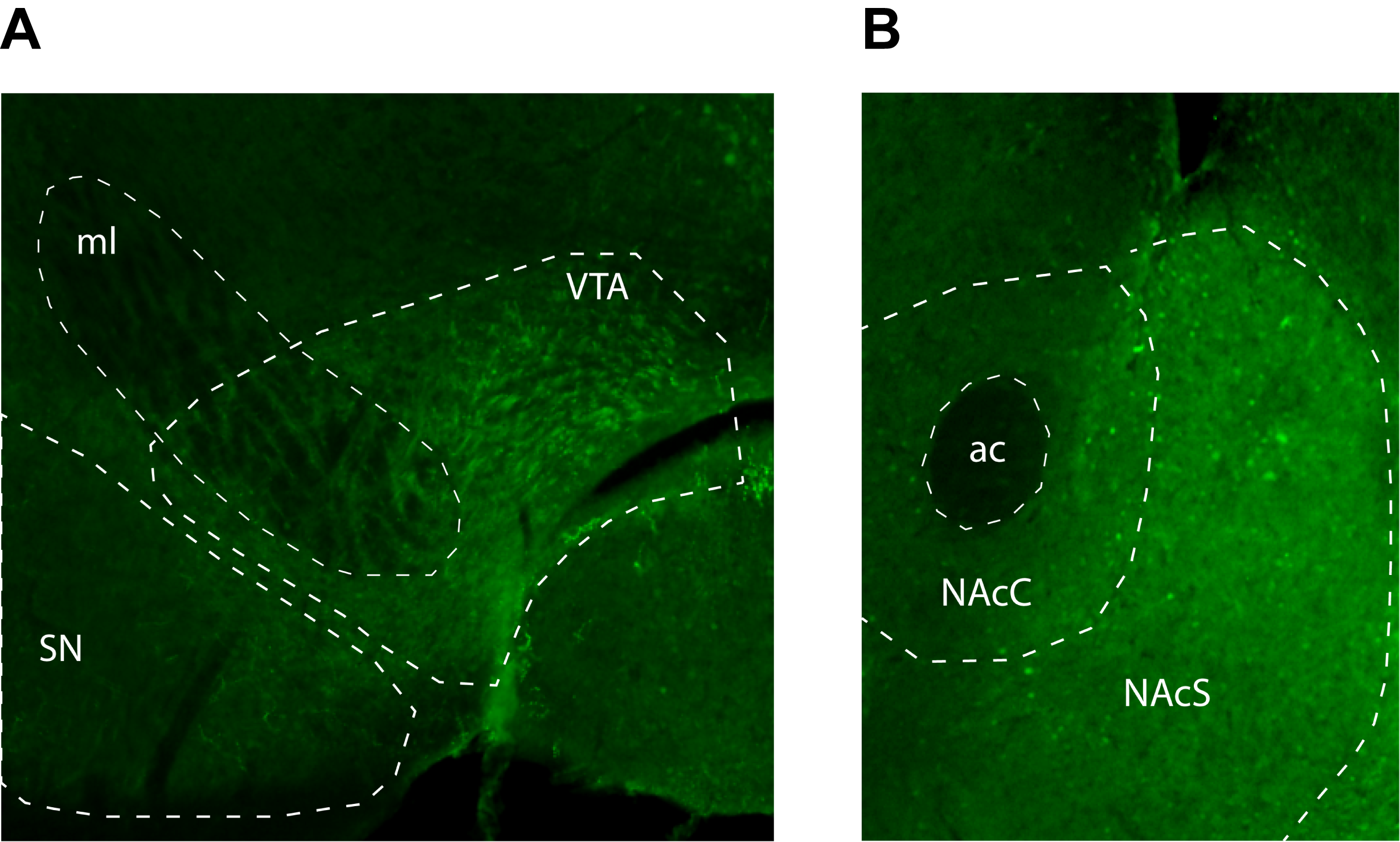

Supplement: Supplementary file 2 — Figure S2 [file 41386_2018_271_MOESM2_ESM.tif]
