# Supplementary material for: Dorsal raphe serotonin neurons inhibit operant responding for reward via inputs to the ventral tegmental area but not the nucleus accumbens: evidence from studies combining optogenetic stimulation and serotonin reuptake inhibition
Source: Neuropsychopharmacology. 2018 Nov 12;44(4):793–804. doi: 10.1038/s41386-018-0271-x (PMC6372654; doi:10.1038/s41386-018-0271-x)
Supplement: Supplementary file 3 — Supplementary Material [file 41386_2018_271_MOESM3_ESM.docx]

**SUPPLEMENTARY MATERIAL**

**Supplementary Methods**

*1.1 In vivo Microdialysis and High Performance Liquid Chromatography*

Acute microdialysis procedures were carried out as previously described (Browne *et al*, 2017) and detailed in Supplementary Methods. Throughout the procedure, mice were maintained under inhaled isoflurane anesthesia (2%) with body temperature held at 37ºC. Mice were mounted on a stereotaxic frame and two burr holes were made in the skull: one above the DRN and one above the NAc. An optical fiber (200 µm core, 0.39 NA, Thorlabs, Newton, NJ) connected to a 473 nm diode-pumped solid laser (Laserglow, Toronto, Canada) was positioned above the DRN (Interaural: A/P -0.8, M/L 0, D/V +3.0; Paxinos and Franklin, 2007). The output of the laser was controlled by a waveform generator (Keysight Technologies, Mississauga, ON, Canada). A microdialysis probe (2mm cuprophane membrane; Scientific Products, Toronto, Canada) was lowered into the left NAc (Bregma: A/P +1.5, M/L -0.7, D/V -5.0 from probe tip; Paxinos and Franklin, 2007), spanning the entire region. The NAc was chosen as a target to sample downstream 5-HT release because it receives dense input from the DRN while also being a large enough region in the mouse brain to accommodate a microdialysis probe. The probe was continuously perfused with aCSF at 1 µl/min using a 1.0 ml gastight syringe and syringe pump (CMA Microdialysis, Holliston, MA). Sampling began 90 minutes following probe insertion. Samples were collected every 10 minutes, and immediately analyzed for 5-HT concentration using high-performance liquid chromatography. Baseline 5-HT concentration was considered stable when 3 consecutive samples varied less than 10% (Baseline).

Measurement of 5-HT levels in dialysate samples was conducted on an analytical system consisting of an Antec Leyden LC110 Alexys HPLC system coupled to a Decade-II electrochemical detection cell and an ALF 105 50x1mm column with C-18 3 µm packing material (ATS Scientific, Burlington, Canada). 5-HT detection potential was 650 mV against an Ag/AgCl electrode. The mobile phase consisted of 50 mM phosphoric acid, 8 mM NaCl, 0.1 mM EDTA, 500 mg/L OSA, and 13% methanol in purified, distilled water. The pH was adjusted to 6.0 and the solution was filtered through a 0.22 µm nylon filter. The flow rate was 55 µL/min. The detection limit for 5-HT was 0.04 nM. Chromatograms were interpreted using Clarity software.

*1.2 Experimental procedures for in vivo microdialysis experiments*

The effects of stimulating DRN 5-HT neurons on downstream 5-HT release was first examined in female ChR2+ mice (n=6). Once baseline extracellular 5-HT concentration was stable, 2.5 Hz photostimulation was applied for 20 min (2 samples) and was terminated for the next 30 min (3 samples). Subsequently, 2.5 Hz photostimulation was applied for 60 min and 5-HT samples were collected throughout (6 samples) to determine whether 5-HT release would continually increase with extended stimulation. In these experiments, baseline 5-HT concentration was reliably detected for all mice, and data is expressed as a percentage of baseline 5-HT concentration.

Changes in NAc 5-HT concentration produced by combining DRN 5-HT neuron photostimulation with citalopram treatment were examined in male ChR2- (n=4) and ChR2+ (n=4) mice. Once baseline 5-HT concentration was stable, mice were treated with 5 mg/kg citalopram and, after 20 min elapsed (2 samples), 2.5 Hz DRN photostimulation was applied for 20 min (2 samples), after which photostimulation was terminated and samples were collected for 80 more min (8 samples). In these experiments, 5-HT levels could not be measured for 6 of the 8 mice at baseline. However, in the first samples following citalopram treatment, 5-HT levels were measured reliably in all animals. Thus, 5-HT levels appeared to be near the detection limit of the HPLC system, which has been observed by others (Sharp et al., 1989), and is likely reflective of low tonic 5-HT release in the NAc. Therefore, baseline for these experiments was set to the detection limit of the HPLC system (0.04 nM).

*2.1 Operant Testing Apparatus*

While in the operant boxes, mice could be tethered to optical patch cables (0.37 NA; Doric Lenses, QC, Canada) which were joined to fiber implants by a ceramic sleeve (Precision Fiber Products, Milpitas, CA). Ceramic sleeves were covered with rubber to prevent light leakage. Patch cables were connected to either a 1-1 optical commutator or a 1-2 splitting optical commutator (0.22 NA; Doric Lenses) through a hole in the top of the operant box. Optical commutators were connected to a 473 nm diode-pumped solid state laser (Laserglow, Toronto, ON, Canada), the output of which was controlled by a waveform generator (Keysight Technologies, Mississauga, ON, Canada). Both the laser and waveform generator were mounted outside of the sound-attenuating chamber.

*2.2 Procedures for measuring operant responding for saccharin*

Prior to surgery, mice were trained to respond for saccharin (0.2% w/v in tap water). Mice were first acclimatized to water restriction for one week and were given access to a bottle containing saccharin in their homecage three times to reduce neophobia in subsequent test phases. Mice were then trained to retrieve saccharin (0.02 ml) from the reward magazine when it was presented. In two 30 minute sessions, the dipper containing saccharin was raised for 8s 60 times according to an RT 30-s schedule. Subsequently, mice were trained to lever press for saccharin. One lever was presented at the beginning of testing, responding on which could result in elevation of the dipper containing saccharin for 5s, after which time the dipper descended. In 40 minute sessions, mice were trained to lever press for saccharin according to a fixed-ratio 1 schedule of reinforcement until they received >30 saccharin presentations in two consecutive sessions. Testing was subsequently completed according to a random ratio 4 (RR4) schedule of reinforcement (1-in-4 chance of reinforcer delivery following response).

Following stabilization of responding for saccharin on the RR4 schedule of reinforcement, mice underwent stereotaxic surgery to receive optical fiber implants. One week following surgery, responding for saccharin was examined in 20 minute sessions with mice tethered to the optical commutator. Mice first received 10 re-training sessions to learn to accommodate the tether inside the operant chamber. During these sessions, no photostimulation was applied. Specific experimental details are outlined below.

*2.3 Stereotaxic surgery*

Mice maintained on inhaled isoflurane anesthesia (2%) received stereotaxic surgery to implant optical fibers. Throughout the procedure, mice were maintained under inhaled isoflurane anesthesia (2%) with body temperature held at 37ºC. Optical fiber implants were constructed from a segment of multimode optical fiber (200 µm core, 0.39 NA; Thorlabs) threaded through a zirconia ferrule (1.25 mm OD; Thorlabs). Mice were mounted on a stereotaxic frame and two burr holes were made in the skull above the DRN, bilaterally above the VTA, or bilaterally above the NAc. Optical fibers implants were positioned above the DRN (unilateral/midline, 0°; -0.8 AP, 0 ML, +3.0 DV from interaural zero), the VTA (bilateral, 10° from midline; -3.4 AP, ±0.5 ML, -4.0 DV from bregma), or the NAc (bilateral, 5° from midline; +1.6 AP, ±0.7 ML, -4.25 DV from bregma) according to Paxinos and Franklin (2007). Ferrules were fixed to the skull using dental cement (RelyX Unicem; 3M, Maplewood, MN). Mice were given one week to recover from surgery.

*2.4 Behavioral effects of optogenetic stimulation of DRN 5-HT neurons*

In water-restricted male ChR2+ (n=10) and ChR2- mice (n=11), responding for saccharin was examined while DRN photostimulation (10 mW, 10 ms pulse width) was applied for the duration of the session. In separate sessions, photostimulation was delivered at frequencies of 1, 5, 10, or 20 Hz. These frequencies were chosen to span the range of 5-HT neuron activity measured during reward-related behavior (Bromberg-Martin et al., 2010, Nakamura et al., 2008). For 20 Hz photostimulation, light pulses were delivered in an ON-OFF-ON pattern with 3s ON and 2s OFF to prevent the development of depolarization block of 5-HT neurons over the 20-min test period (McDevitt et al., 2014). Based on the outcome of these experiments, we then examined the effect of DRN photostimulation in combination with systemic 5-HT reuptake blockade using the SSRI citalopram. ChR2+ and ChR2- mice received intraperitoneal injections of either 5 or 10 mg/kg of citalopram, or its vehicle, and responding for saccharin was examined with 2.5 Hz DRN photostimulation applied throughout the session. The 2.5 Hz frequency was chosen to approximate the average firing rate of DRN 5-HT neurons without citalopram on board (Jacobs and Azmitia, 1992). This testing procedure was also repeated with 5 and 10 Hz DRN photostimulation.

Following tests of responding for saccharin, the effects of optogenetic stimulation of DRN 5-HT neurons combined with citalopram treatment on locomotor activity was examined. Following injection of 5 or 10 mg/kg citalopram or its vehicle, activity tests were conducted in 20 minute sessions with 2.5 Hz DRN photostimulation applied throughout.

Following tests of locomotor activity, measures of serotonin syndrome were obtained based on (Haberzettl et al., 2013) using a between-subjects design. ChR2- and ChR2+ mice were treated with 5 mg/kg citalopram and behavior was recorded for a 20-min period in which 2.5 Hz DRN photostimulation was applied throughout. See section 2.7 for details.

*2.5 Behavioral effects of optogenetic stimulation of 5-HT terminals in the VTA*

Responding for saccharin was examined in water-restricted, male ChR2- (n=5) and ChR2+ (n=7) mice which received bilateral optical fiber implants targeting the VTA. The effects of photostimulation alone (5 mW, 10 ms pulse width) was first examined in ChR2+ mice. On alternating test days, mice received either photostimulation at different frequencies (5, 10, or 20 Hz) for the duration of testing, or no photostimulation. Subsequently, both ChR2- and ChR2+ mice were tested with 5 mg/kg citalopram alone or its vehicle, followed by the combination of citalopram (or its vehicle) and 5 Hz photostimulation of the VTA applied throughout test sessions.

Following tests of responding for saccharin, the effects of optogenetic stimulation of 5-HT input to the VTA on locomotor activity were examined in 20 minute sessions. ChR2- and ChR2+ mice were tested with 5 mg/kg citalopram alone or its vehicle, followed by the combination of citalopram (or its vehicle) and 5 Hz photostimulation of the VTA applied throughout test sessions.

*2.6 Behavioral effects of optogenetic stimulation of 5-HT terminals in the NAc*

Responding for saccharin was examined in water-restricted male ChR2- (n=8) and ChR2+ (n=8) mice which received bilateral photostimulation of the NAc. The effects of photostimulation alone (5 mW, 10 ms pulse width) was first examined in ChR2+ mice. On alternating test days, mice received either photostimulation at different frequencies (5, 10, or 20 Hz) for the duration of testing, or no photostimulation. Subsequently, both ChR2- and ChR2+ mice were tested with 5 mg/kg citalopram alone or its vehicle, followed by the combination of citalopram (or its vehicle) and 5 Hz photostimulation of the NAc applied throughout test sessions.

Following tests of responding for saccharin, the effects of optogenetic stimulation of 5-HT input to the NAc on locomotor activity were examined in 20 minute sessions. ChR2- and ChR2+ mice were tested with 5 mg/kg citalopram alone or its vehicle, followed by the combination of citalopram (or its vehicle) and 5 Hz photostimulation of the NAc applied throughout test sessions.

*2.7 Procedures for measuring serotonin syndrome*

Serotonin syndrome measurements were scored at half the frame rate of the original video. Continuous measures were: flat body posture, tremor, straub tail, forepaw treading, backward walking, head weaving. Discrete measures were: head twitches, rearing, and fecal boli. Scores were taken for 180s at 5-min intervals, beginning at the start of testing. Continuous measurements were scored as either present (1) or absent (0) for each 180s period for each animal. Total scores were generated by summing present or absent across all four sampling periods, generating a score of 0-4 for each animal which was averaged for each group.

*3.1 Histological procedures*

Mice were transcardially perfused with PBS (7.4 pH) followed by 4% paraformaldehyde. Brains were extracted and postfixed overnight with 4% paraformaldehyde at 4°C and cryoprotected with 30% sucrose in PBS. Coronal sections (40 um thickness) were collected on a cryostat. Free-floating immunohistochemistry was performed according to standard procedures. Sections were blocked with 5% donkey serum in 0.1% PBS-T for 1h at room temperature. Subsequently, sections were co-incubated with mouse monoclonal anti-tryptophan hydroxylase-2 (1:500; Sigma-Aldrich) and chicken anti-GFP (1:1000; Abcam) primary antibodies for 48 h at 4 °C. Sections were then co-incubated with donkey anti-mouse Alexa 594 (1:1000; Invitrogen) and donkey anti-chicken Alexa 488 (1:1000; Jackson Immunoresearch) secondary antibodies for 2h at room temperature. Sections were mounted and imaged on a confocal laser scanning microscope (Olympus).

**Figure S1.** Effects of combining 5 mg/kg citalopram and optogenetic stimulation (10 mW, 10 ms pulses) of DRN 5-HT neurons at 5 and 10 Hz photostimulation frequencies on responding for saccharin (from mice in Figures 1I and 3). Compared to ChR2- mice (n=10, white symbols), ChR2+ mice (n=11, green symbols) showed a greater reduction in responding for saccharin at both the 5 Hz (A; Group×Dose×Session Time: *F*_(6,114)_=11.45, *p*<0.0001) and 10 Hz (B; Group×Dose×Session Time: *F*_(6,114)_=13.79, *p*<0.0001) photostimulation frequencies. Data are expressed as mean (± SEM).

**Figure S2**. Representative coronal sections from a ChR2+ mouse showing density of ChR2-EYFP expression in 5-HT terminal fields within the ventral tegmental area (VTA; panel A) and the nucleus accumbens (NAc; panel B) relative to surrounding regions. ml, medial lemniscus; SN, substantia nigra; ac, anterior commissure; NAcC, nucleus accumbens core; NAcS, nucleus accumbens shell.

**Table S1** Membrane properties of DRN 5-HT neurons recorded in brain slices from ChR2+ mice at baseline and in the presence of 1 µM citalopram. Data are expressed as mean ± S.E.M. Independent samples t-tests, * denotes *p*<0.05.

| Condition | Resting Membrane Potential (mV) | Spike Threshold  (mV) | Spike Amplitude  (mV) | Input Resistance  (MΩ) |
| --- | --- | --- | --- | --- |
| Baseline  (n = 13) | - 68 ± 3 | - 49 ± 1 | 68 ± 2 | 521 ± 44 |
| 1 μM Citalopram  (n = 8) | - 81 ± 4 | - 47 ± 2 | 68 ± 6 | 340 ± 68 |
| *t*-test (*P* value) | 0.02* | 0.3 | 0.9 | 0.03* |

**Table S2.** Serotonin syndrome-like responses in ChR2- and ChR2+ mice under conditions of optogenetic stimulation of DRN 5-HT neurons (2.5 Hz, 10 mW, 10 ms pulse width) combined with systemic 5 mg/kg citalopram treatment. Data are expressed as mean ± SEM.

| Response | Group |  |  |
| --- | --- | --- | --- |
|  | ChR2- | ChR2+ | *t-test*  *p-value* |
| *Average scores:* |  |  |  |
| Flat body posture | 0.4 ± 0.4 | 0.7 ± 0.5 | 0.614 |
| Tremor | 0.0 ± 0.0 | 0.0 ± 0.0 | - |
| Straub tail | 0.0 ± 0.0 | 0.0 ± 0.0 | - |
| Forepaw treading | 0.0 ± 0.0 | 0.0 ± 0.0 | - |
| Backward walking | 0.0 ± 0.0 | 0.0 ± 0.0 | - |
| Head weaving | 0.0 ± 0.0 | 0.0 ± 0.0 | - |
| Head twitches [n] | 2.3 ± 0.7 | 1.9 ± 0.5 | 0.653 |
| Rearing [n] | 43.5 ± 4.2 | 43.4 ± 4.9 | 0.995 |
| Fecal Boli [n] | 2.1 ± 0.3 | 3.0 ± 0.5 | 0.148 |
