# Supplementary material for: Dorsal raphe serotonin neurons inhibit operant responding for reward via inputs to the ventral tegmental area but not the nucleus accumbens: evidence from studies combining optogenetic stimulation and serotonin reuptake inhibition
Source: Neuropsychopharmacology. 2018 Nov 12;44(4):793–804. doi: 10.1038/s41386-018-0271-x (PMC6372654; doi:10.1038/s41386-018-0271-x)
Supplement: Supplementary file 4 — Supplementary Material ReadMe File [file 41386_2018_271_MOESM4_ESM.docx]

Supplementary Information “Read-Me”

Supplementary methods: Detailed description of methodology as referenced in the manuscript.

Table S1: Data table presenting the *ex vivo* electrophysiological properties of dorsal raphe nucleus serotonin neurons under baseline conditions, following optogenetic stimulation, and following bath application of citalopram (1 um). Significant effects are marked with an asterisk.

Table S2: Data table presenting the effects of combined optogenetic stimulation and citalopram treatment on measures of the serotonin syndrome. No significant effects were observed.

Figure S1: Figure presenting additional experiments from Figure 4. The effects of citalopram combined with 5 Hz and 10 Hz optogenetic stimulation on responding for saccharin are presented.

Figure S2: Figure presenting coronal sections of the VTA and NAc from ChR2+ mice showing EYFP-positive 5-HT terminal fields.
